# Draft genome sequences of *Tenacibaculum finnmarkense* isolates from diseased Atlantic salmon (*Salmo salar*) in Chilean farms

**DOI:** 10.1128/mra.00385-25

**Published:** 2025-07-18

**Authors:** Ruben Avendaño-Herrera, Mónica Saldarriaga-Córdoba

**Affiliations:** 1 Universidad Andrés Bello, Laboratorio de Patología de Organismos Acuáticos y Biotecnología Acuícola, Facultad de Ciencias de la Vida 28087, Viña del Mar, Chile; 2 Centro FONDAP, Interdisciplinary Center for Aquaculture Research (INCAR), Universidad Andrés Bello 28087, Viña del Mar, Chile; 3 Centro de Investigación Marina Quintay (CIMARQ), Universidad Andrés Bello, Quintay, Chile; 4 Escuela de Medicina Veterinaria, Centro de Investigación en Recursos Naturales y Sustentabilidad, Universidad Bernardo O'Higgins 28042, Santiago, Chile; University of Southern California, Los Angeles, California, USA

**Keywords:** tenacibaculosis, fish pathogen, salmonids

## Abstract

We report the draft genome sequences of *Tenacibaculum finnmarkense* isolates from diseased Atlantic salmon (*Salmo salar*) in Chile, providing initial insights into the genomics of this pathogen. Currently, limited data exist on Chilean isolates to aid in the development of genome-based diagnostic tools, typing methods, and/or prevention strategies for aquaculture applications.

## ANNOUNCEMENT

*Tenacibaculum finnmarkense* was first isolated in 2016 from diseased Atlantic salmon (*Salmo salar*) in Finnmark, Norway (1). Taxonomic validation did not occur until 2020, when a subdivision into genomovars *finnmarkense* and *ulcerans* was proposed (2). This gram-negative, obligate aerobe forms yellow-pigmented colonies, exhibits gliding motility, and grows at 2°C–20°C in marine salts (2). The AYD7486TD isolate is the only analyzed genome in Chile (3). We present the draft genomes of isolates LB-P5 and P3-BQB, recovered from the skin lesions and gills of diseased Atlantic salmon during tenacibaculosis outbreaks in July 2017 (42°54′59″S, 72°42′30″W) and June 2018 (45°01′47″S, 73°37′29″W), respectively, at separate farming sites. These isolates were obtained as dominant colonies after streaking fish samples onto Marine Agar 2216 (BD Difco) plates and incubating them at 18°C for 1 week.

For genomic sequencing, both isolates were cultured on Marine Agar 2216 at 18°C for 48 h. Genomic DNA was purified (NucleoSpin Soil Kit; Macherey-Nagel), quantified (NanoDrop Lite Spectrophotometer; Thermo Fisher Scientific Inc.), and, afterward, sequenced by SeqCenter (PA, USA). Library preparation was performed using the Illumina DNA Prep Kit with IDT 10 bp UDI indices and sequenced on Illumina NextSeq 2000, generating 2 × 151 bp paired-end reads. Prior to *de novo* genome assembly, raw Illumina sequencing data underwent quality control (FastQC version 0.12.1; https://www.bioinformatics.babraham.ac.uk/projects/fastqc/). Next-generation sequencing reads were pre-processed (Geneious Prime version 2024.0.5) (4): (i) pairing reads using the “set paired reads” function (insert size = 350 bp); (ii) trimming poor-quality bases from read ends (BBDuk Trimmer plugin version 38.84; reads <50 bp and with a quality score <30 were removed), with paired-read overlaps trimmed to ensure complete adapter removal; (iii) normalizing coverage by down-sampling reads in high-depth genome areas with the “error correct” and “normalize reads” functions (BBNorm version 38.84) (5); and (iv) removing duplicate reads (Dedupe plugin). Assemblies (Spades version 4.0.0) (6) were evaluated for quality (QUAST version 5.2.0) and completeness (CheckM version 1.2.2) (7, 8). Genomes were annotated using RAST server version 2.0 (9) (https://figshare.com/s/5f65c20fb8995e0eff83) and Prokaryotic Genome Annotation Pipeline version 6.9 (19 March 2025). All software used the default settings.

Genome assemblies (Table 1) contained 56 (LB-P5) and 55 (P3-BQB) contigs; 2,752,994 and 2,800,552 bp; and a G + C content of ∼31.0%. Filtered reads were mapped using SOAP (10) to the bacterial plasmid database (11). No plasmids were found in the two isolates analyzed. Both strains were *T. finnmarkense* (JSpeciesWS-Taxonomic Thresholds [12] and GTDB-Tk version 2.4.0 [13]); the closest reference strain was the *ulcerans* genomovar (accession number NZ_OENE01000000), with an average nucleotide identity of 98.64% for LB-P5 and 98.75% for P3-BQB.

**TABLE 1:** *Tenacibaculum finnmarkense* isolate genomes released to the NCBI

Strain	SRA	Accession no.	No. of reads^*a*^	Total length (bp)^*b*^	Completeness^*c*^	Genome coverage	Contamination^*c*^	Contigs^*b*^	%GC^*b*^	*N*_50_ (kb)^*b*^	Genes^*d*^	CDS^*d*^
LB-P5	SRR32778091	JBMFZR000000000	5,517,150/ 2,694,444	2,752,994	80.35	100×	4.52%	56	31.06	190.7	2,403	2,352
P3-BQB	SRR32778090	JBMFZS000000000	10,437,554/ 2,749,628	2,800,552	79.98	100×	2.36%	55	31.01	190.5	2,473	2,416

^*a*^ Number of original reads/normalized read depth using BBNorm.

^*b*^ Determined using SPAdes version 4.0.0.

^*c*^ Determined by CheckM version 1.2.3.

^*d*^ Determined using RAST version 2.0 and PGAP version 6.9.

This study provides initial insights into the genomic characteristics of *T. finnmarkense* genomovar *ulcerans* strains isolated from Chilean tenacibaculosis outbreaks in farmed Atlantic salmon, offering genomic details on potential virulence that require further biochemical and physiological studies.

## Data Availability

This whole-genome shotgun project has been deposited in DDBJ/ENA/GenBank under the accession numbers JBMFZR000000000 (LB-P5)–JBMFZS000000000 (P3-BQB). BioProject ID PRJNA1238331 is publicly available (https://www.ncbi.nlm.nih.gov/bioproject/?term=PRJNA1238331) and contains BioSample SAMN47468122–SAMN47468123 and SRA records (SRR32778090-P3-BQB; SRR32778091-LB-P5).
